# Feynman Paths and Weak Values

**DOI:** 10.3390/e20050367

**Published:** 2018-05-14

**Authors:** Robert Flack, Basil J. Hiley

**Affiliations:** Department of Physics and Astronomy, University College London, Gower Street, London WC1E 6BT, UK

**Keywords:** Feynman paths, weak values, Bohm theory

## Abstract

There has been a recent revival of interest in the notion of a ‘trajectory’ of a quantum particle. In this paper, we detail the relationship between Dirac’s ideas, Feynman paths and the Bohm approach. The key to the relationship is the weak value of the momentum which Feynman calls a transition probability amplitude. With this identification we are able to conclude that a Bohm ‘trajectory’ is the average of an ensemble of actual individual stochastic Feynman paths. This implies that they can be interpreted as the mean momentum flow of a set of individual quantum processes and not the path of an individual particle. This enables us to give a clearer account of the experimental two-slit results of Kocsis et al.

## 1. Introduction

One of the basic tenets of quantum mechanics is that the notion of a particle trajectory has no meaning. The established view has been unambiguously defined by Landau and Lifshitz [1]: “In quantum mechanics there is no such concept as the path of a particle”. This position was not arrived at without an extensive discussion going back to the early debates of Bohr and Einstein [2], the pioneering work of Heisenberg [3] and many others [4].

Yet Kocsis et al. [5] have experimentally determined an ensemble of what they call ‘photon trajectories’ for individual photons traversing a two-slit interference experiment. The set of trajectories, or what we will call flow-lines, they construct is very similar in appearance to the ensemble of Bohmian trajectories calculated by Philippidis et al. [6]. Mahler et al. [7] have gone further and claimed that their new experimental results provide evidence in support of Bohmian mechanics. However such a claim cannot be correct because Bohmian mechanics is based on the Schrödinger equation which holds only for non-relativistic particles with non-zero rest mass, whereas photons are relativistic, having zero rest mass.

The flow-lines are calculated from experimentally determined weak values of the momentum operator, a notion that was introduced originally by Aharonov et al. [8] for the spin operator. When examined closely, the momentum weak value is the Feynman transition probability amplitude (TPA) [9]. In fact, Schwinger [10] explicitly writes the TPA of the momentum in exactly the same form as the weak value. Recall that the TPA involving the momentum operator plays a central role in the discussion of the path integral method, an approach that was inspired by an earlier paper of Dirac [11] who was interested in developing the notion of a ‘quantum trajectory’.

Weak values are in general complex numbers, as are TPAs. The real part of the momentum weak value is the local momentum, sometimes known as the Bohm momentum. The imaginary part turns out to be the osmotic momentum introduced by Nelson [12] in his stochastic derivation of the Schrödinger equation. In this paper, we will show how the weak value of momentum, Feynman paths and the Bohm trajectories are related enabling us to give a different meaning to the flow-lines constructed in experiments of the type carried out by Kocsis et al. [5] and Mahler et al. [7].

Feynman [9] also shows that in his approach the usual expression for the kinetic energy becomes infinite unless one introduces a small fluctuation in the mass of the particle. We will show that this is equivalent to introducing the quantum potential, a new quality of energy that appears in the real part of the Schrödinger equation under polar decomposition of the wave function [13].

## 2. Dirac’s Notion of a Quantum Trajectory

### 2.1. Dirac Trajectories

To make the context of our discussion clear, we will begin by drawing attention to an early paper by Dirac [11] who attempted to generalise the Heisenberg algebraic approach through his unique bra-ket notation, not as elements in a Hilbert space, but as elements of a non-commutative algebra. In this approach the operators of the algebra are functions of time. Dirac argued that to get round the difficulties presented by a non-commutative quantum algebra, strict attention must be paid to the time-order of the appearance of elements in a sequence of operators.

In the non-relativistic limit, operators at different times always commute. (In this paper, we will, for simplicity, only consider the non-relativistic domain. Dirac himself shows how the ideas can be extended to the relativistic domain.) This means that a time ordered sequence of position operators can be written in the form,

(1) $$\langle x_{t}|x_{t_{0}}\rangle =\int \ldots \int \langle x_{t}|x_{t_{j}}\rangle dx_{j}\langle x_{t_{j}}|x_{t_{j-1}}\rangle \ldots \langle x_{t_{2}}|x_{t_{1}}\rangle dx_{1}\langle x_{t_{1}}|x_{t_{0}}\rangle .$$

This breaks the TPA, $\langle x_{t}|x_{t_{0}}\rangle$, into a sequence of adjacent points, each pair connected by an infinitesimal TPA. Dirac writes “...one can regard this as a trajectory ...and thus makes quantum mechanics more closely resemble classical mechanics”.

In order to analyse the sequence in Equation (1) further, Dirac assumed that for a small time interval Δt=ϵ, we can write

(2) $${\langle x|x^{\prime }\rangle }_{\epsilon }=\exp[iS_{\epsilon }\left(x,x^{\prime }\right)/\hbar ]$$

where we will take $S_{\epsilon }\left(x,x^{\prime }\right)$ to be a real function in the first instance. Then Dirac [14] shows that

(3) $$p_{\epsilon }^{\prime }\left(x,x^{\prime }\right)=\langle x|{\hat{P}}^{\prime }{|x^{\prime }\rangle }_{\epsilon }=i\hbar \nabla_{x^{\prime }}{\langle x|x^{\prime }\rangle }_{\epsilon }=-\nabla_{x^{\prime }}S_{\epsilon }\left(x,x^{\prime }\right){\langle x|x^{\prime }\rangle }_{\epsilon }$$

and

(4) $$p_{\epsilon }\left(x,x^{\prime }\right)=\langle x|\hat{P}{|x^{\prime }\rangle }_{\epsilon }=-i\hbar \nabla_{x}{\langle x|x^{\prime }\rangle }_{\epsilon }=\nabla_{x}S_{\epsilon }\left(x,x^{\prime }\right){\langle x|x^{\prime }\rangle }_{\epsilon }.$$

Here $\hat{P}$ is the momentum operator. The remarkable similarity of these objects to the canonical momentum appearing in the classical Hamilton-Jacobi theory should be noted, a fact of which Dirac was well aware. They are also the canonical momenta appearing in the real part of the Schrödinger equation under polar decomposition of the wave function exploited by Bohm [13] who identified the momentum with the gradient of the phase of the wave function.

In an earlier paper, Dirac [11] did not specify how $S_{\epsilon }\left(x,x^{\prime }\right)$ could be determined. It was Feynman [9] who later identified its relation to the classical Lagrangian $L(\dot{x},x,t)$ through the relation

(5) $$\begin{array}{c} S_{tt^{\prime }}\left(x,x^{\prime }\right)=\mathrm{Min}\int_{t^{\prime }}^{t}L\left(\dot{x},x,t\right)dt. \end{array}$$

However, this Lagrangian determines the classical path, so using the exponent of the classical action seems puzzling. Is there a mathematical explanation for such a choice? The answer is ‘yes’ and is discussed in Guillemin and Sternberg [15]. The essential reason for this lies in the relation between the symmetry group, in this case the symplectic group, and its covering group. Exploiting this structure, de Gosson and Hiley [16] have shown in detail how it is possible to mathematically ‘lift’ classical trajectories onto this covering space. It is from this structure that the wave properties emerge. The lift is achieved by exponentiating the classical action, namely using $\exp[iS_{\epsilon }\left(x,x^{\prime }\right)]$. It is the existence of this structure that the close relation between the Dirac quantum ‘trajectories’ and the de Broglie-Bohm ‘trajectories’ first calculated by Philippidis et al. [6] emerges. We will bring out this relationship in the rest of this paper.

### 2.2. The Feynman Propagator

Equation (5) allows us to write the propagator in the well known form

$$\begin{array}{c} K\left(x,x^{\prime }\right)=\int_{x^{\prime }}^{x}e^{iS(x,x^{\prime })}Dx^{\prime } \end{array}$$

where the integral is taken over all paths connecting $x^{\prime }$ to *x*. We have written $Dx^{\prime }$ for $\frac{dx_{0}}{A},\ldots ,\frac{dx_{j-1}}{A}$ where $\left(x_{0},x_{1},\ldots ,x_{j-1}\right)$ are points on the path and *A* is the normalising factor introduced by Feynman. Clearly here $S(x,x^{\prime })$ is real.

For a free particle with mass *m*, we have $L=m{\dot{x}}^{2}/2$ and one can show that

(6) $$\begin{array}{c} K_{tt^{\prime }}\left(x,x^{\prime }\right)=\frac{1}{A}\exp\left[\frac{im{\left(x-x^{\prime }\right)}^{2}}{2\hbar (t-t^{\prime })}\right] \end{array}$$

where $A={\left(\frac{2\pi i(t-t^{\prime })}{m}\right)}^{1/2}$. With this propagator, Feynman was able to derive the Schrödinger equation by assuming the underlying paths were continuous and differentiable.

However if we examine the terms ${\langle x|x^{\prime }\rangle }_{\epsilon }$ for ϵ→0, we find the curves, although continuous, are non-differentiable. To show this let us introduce the TPA of a function F(x,t) defined by

$$\begin{array}{c} \langle \phi_{t}\left|F\right|\psi_{t^{\prime }}\rangle_{S}={\mathrm{Lim}}_{\epsilon \to 0}\int \ldots \int \phi^{*}\left(x,t\right)F\left(x_{0},x_{1},\ldots ,x_{j}\right)\\ \times \exp\left[\frac{i}{\hbar }\sum_{k=0}^{j-1}S\left(x_{k+1},x_{k}\right)\right]\psi \left(x^{\prime },t^{\prime }\right)Dx\left(t\right). \end{array}$$

Here D is now written as $Dx\left(t\right)=\frac{dx_{0}}{A}\ldots \frac{dx_{j-1}}{A}dx_{j}$.

These TPAs can be evaluated by using functional derivatives. In fact, the average of the functional derivative of a function F(x,t) is given by

(7) $$\begin{array}{c} {\langle \frac{\delta F}{\delta x(s)}\rangle }_{S}=-\frac{i}{\hbar }{\langle F\frac{\delta S}{\delta x(s)}\rangle }_{S} \end{array}$$

at the point x(s) on the path x(t). In the case of the specific integral

$$\begin{array}{c} \int \frac{\partial F}{\partial x_{k}}\exp\left[\left(i/\hbar \right)S\left(x\left(t\right)\right)\right]Dx\left(t\right), \end{array}$$

Equation (7) can be written in the form

$$\begin{array}{c} {\langle \frac{\partial F}{\partial x_{k}}\rangle }_{S}=-\frac{i}{\hbar }{\langle F\frac{\partial S}{\partial x_{k}}\rangle }_{S}. \end{array}$$

Feynman notes that the quantities in this expression need not be observables, nevertheless the equivalence is true [17].

Let us now consider three adjacent points $x_{k-1},x_{k},x_{k+1}$, each separated by a small time difference ϵ, we have

$$\begin{array}{c} -\frac{\hbar }{i}{\langle \frac{\partial F}{\partial x_{k}}\rangle }_{S}={\langle F\left[\frac{\partial S(x_{k+1},x_{k})}{\partial x_{k}}+\frac{\partial S(x_{k},x_{k-1})}{\partial x_{k}}\right]\rangle }_{S}. \end{array}$$

This equation is correct to zero and first order in ϵ. If we choose the action for a particle moving in a potential *V*, we have

$$\begin{array}{c} S\left(x,x^{\prime }\right)=\left[\frac{m{\left(x-x^{\prime }\right)}^{2}}{2\epsilon }\right]-\epsilon V\left(x,x^{\prime }\right). \end{array}$$

Then at the point $x_{k}$ this gives us

$$\begin{array}{c} -\frac{\hbar }{i}{\langle \frac{\partial F}{\partial x_{k}}\rangle }_{S}=\langle F\left[-m\left(\frac{x_{k+1}-x_{k}}{\epsilon }-\frac{x_{k}-x_{k-1}}{\epsilon }\right)-\epsilon \frac{\partial V}{\partial x_{k}}\left(x_{k}\right)\right]\rangle . \end{array}$$

If *F* is unity and we divide by ϵ we get

(8) $$\begin{array}{c} 0=\langle \frac{1}{\epsilon }\left[-m\left(\frac{x_{k+1}-x_{k}}{\epsilon }-\frac{x_{k}-x_{k-1}}{\epsilon }\right)-\frac{\partial V}{\partial x_{k}}\left(x_{k}\right)\right]\rangle . \end{array}$$

If we follow Feynman and call $(x_{k+1}-x_{k})/\epsilon$ a ‘velocity’, then this equation gives the ‘average’ over an ensemble of individual velocities. It is the *quantum equivalent* of Newton’s second law of motion; the potential *V* at $x_{k}$ gives rise to a force which changes the incoming momentum $m(x_{k}-x_{k-1})/\epsilon$ to the outgoing momentum $m(x_{k+1}-x_{k})/\epsilon$. Notice to order ϵ, no extra term corresponding to the quantum potential appears. de Gosson and Hiley [18] have shown in a detailed analysis that this is to be expected.

These paths are reminiscent of Brownian motion, a characteristic feature of which is the appearance of two ‘derivatives’ at $x_{k}$, a ‘forward’ and a ‘backward’ derivative, illustrating the non-differentiable nature of the path. In this paper, we need not discuss the precise nature of these paths to arrive at our conclusion. It is sufficient for us to note that the substructure of a quantum process is *certainly not classical*. In passing we should also note that the ‘velocities’, being of order ${\left(\hbar /m\epsilon \right)}^{1/2}$, diverge as ϵ→0 and therefore, in Feynman’s terms, are not observables.

### 2.3. TPAs Involving the Momentum

In 1974 Hirschfelder [19,20] introduced a quantity $\psi {\left(x,t\right)}^{-1}\hat{p}\psi \left(x,t\right)$, which he called a ‘sub-observable’ as he could see no way of measuring it directly, although integrating it over the whole of configuration space gave the measurable expectation value. Using the polar form of the wave function, ψ(x,t)=R(x,t)exp[iS(x,t)/ℏ], this ‘sub-observable’ is the weak value of the momentum operator which can be written in the form

(9) $$\begin{array}{c} \psi {\left(x,t\right)}^{-1}\hat{p}\psi \left(x,t\right)=\frac{\langle x|\hat{p}|\psi \left(t\right)\rangle }{\langle x|\psi (t)\rangle }=m\left[v_{B}\left(x,t\right)-iv_{O}\left(x,t\right)\right], \end{array}$$

where explicitly $v_{B}\left(x,t\right)=\nabla S\left(x,t\right)/m$ is the local Bohm velocity and $v_{O}\left(x,t\right)=\nabla R\left(x,t\right)/mR\left(x,t\right)$ is the localising osmotic velocity, originally introduced by Nelson [12] in a stochastic theory. The meaning of these velocities is discussed in more detail in Bohm and Hiley [21]. Much later Hiley [22] showed exactly how these expressions emerged directly from the weak value of the momentum operator. It should be noted that weak values are essentially TPAs of the type considered by Feynman [9] and Schwinger [23].

In the spirit of Schwinger [10], where he argues that “the quantum dynamical laws will find their proper expression in terms of the transformation functions” that is TPAs, we can introduce *two* momentum TPAs, $\langle x|\vec{P}|\psi \left(t\right)\rangle$ and $\langle \psi \left(t\right)|\overset{\leftarrow }{P}|x\rangle$ where $\vec{P}=-i\hbar \vec{\nabla }$ and $\overset{\leftarrow }{P}=i\hbar \overset{\leftarrow }{\nabla }$. Notice by placing the arrows over the momentum operators, we are emphasising the distinction between left and right multiplication and it is this distinction that is equivalent to the forward and backward derivatives. In fact we may identify

$$\begin{array}{c} \langle X|\vec{P}|\psi \left(t^{\prime }\right)\rangle =\langle X|\vec{P}|x^{\prime }\rangle \psi \left(x^{\prime },t^{\prime }\right)=-i\lim_{\left(x^{\prime }\to X\right)}\frac{\psi \left(X\right)-\psi \left(x^{\prime }\right)}{\left(X-x^{\prime }\right)} \end{array}$$

with the forward derivative at *X*, a point that lies between $x^{\prime }$ and *x*.

$$\begin{array}{c} \langle \psi \left(t\right)|\overset{\leftarrow }{P}|X\rangle =\psi^{*}\left(x,t\right)\langle x|\overset{\leftarrow }{P}|X\rangle =i\lim_{\left(X\to x\right)}\frac{\psi^{*}\left(x\right)-\psi^{*}\left(X\right)}{\left(x-X\right)} \end{array}$$

corresponds to the backward derivative. Note that the words ‘forward’ and ‘backward’ here have nothing to do with time order.

If we again evaluate these TPAs using ψ=Rexp(iS/ℏ), we find

(10) $$\begin{array}{c} \frac{1}{2}\left[\frac{\langle x|\vec{\hat{P}}|\psi \left(t\right)\rangle }{\langle x|\psi (t)\rangle }+\frac{\langle \psi \left(t\right)|\overset{\leftarrow }{\hat{P}}|x\rangle }{\langle \psi (t)|x\rangle }\right]=\nabla S\left(x,t\right)=P_{B}\left(x,t\right), \end{array}$$

and

(11) $$\begin{array}{c} \frac{1}{2i}\left[\frac{\langle x|\vec{\hat{P}}|\psi \left(t\right)\rangle }{\langle x|\psi (t)\rangle }-\frac{\langle \psi \left(t\right)|\overset{\leftarrow }{\hat{P}}|x\rangle }{\langle \psi (t)|x\rangle }\right]=\frac{\nabla R(x,t)}{R(x,t)}=P_{O}\left(x,t\right). \end{array}$$

Notice how the sums and differences of the left/right operators produce real values.

We can immediately connect these results with those of Dirac [11] if, in Equations (3) and (4), we replace the real value of $S_{\epsilon }\left(x,x^{\prime }\right)$ by a complex value which we will write as $S_{\epsilon }^{\prime }\left(x,x^{\prime }\right)=S_{\epsilon }\left(x,x^{\prime }\right)-i\ln R_{\epsilon }\left(x,x^{\prime }\right)$. In this case, we find

(12) $$p_{\epsilon }^{\prime }\left(x,x^{\prime }\right)=-\nabla_{x^{\prime }}S_{\epsilon }\left(x,x^{\prime }\right)-i\frac{\nabla_{x^{\prime }}R_{\epsilon }\left(x,x^{\prime }\right)}{R_{\epsilon }\left(x,x^{\prime }\right)}$$

and

(13) $$p_{\epsilon }\left(x,x^{\prime }\right)=\nabla_{x}S_{\epsilon }\left(x,x^{\prime }\right)-i\frac{\nabla_{x}R_{\epsilon }\left(x,x^{\prime }\right)}{R_{\epsilon }\left(x,x^{\prime }\right)}.$$

Notice also the connection with the classical relations obtained in Equations (3) and (4).

### 2.4. The Relation between Weak Values and TPAs

In the previous two sections, we have shown how TPAs of the form $\langle \phi_{t}|\hat{F}|\psi_{t^{\prime }}\rangle$ arise from some underlying non-differentiable process. The original assumption was that these quantities could not be investigated experimentally. However starting from a different perspective, the notion of a weak value, introduced by Aharonov, Albert and Vaidman [8], allows us to experimentally measure these quantities.

A weak value of an operator $\hat{F}$ is defined by

$$\begin{array}{c} {\langle \hat{F}\rangle }_{w}=\frac{\langle \phi_{t}|\hat{F}|\psi_{t^{\prime }}\rangle }{\langle \phi_{t}|\psi_{t^{\prime }}\rangle }. \end{array}$$

Clearly these weak values are Feynman TPAs. Using the suggestions of Leavens [24] and Wiseman [25], Kocsis et al. [5] have actually measured the weak value of the transverse momentum in an optical two-slit experiment and as a result have constructed what they called *photon ‘trajectories’*. We refer to their paper to explain the details of how this is done.

Unfortunately photons cannot be treated as particles that satisfy the Schrödinger equation. They have zero rest mass and are excitations of the electromagnetic field. Nevertheless this does not invalidate the notion of a momentum flow line; the question remains “How are we to understand these flow lines?” Flack and Hiley [26] have shown that if we generalise the Bohm approach to include the electromagnetic field [27], each flow line emerges as the locus of a weak Poynting vector.

To connect with the non-relativistic approach we are discussing in this paper, we need to use atoms. Indeed experiments are being developed at UCL to measure weak values of spin and momentum, ${\langle \hat{p}\rangle }_{w}$, for helium atoms [28] and argon atoms [29] respectively. The experimental details can be found in these references. In this paper, we will clarify further the relation between the Feynman paths and weak values.

## 3. Weak Values Are Weighted TPAs

### 3.1. Flow Lines Constructed from Weak Values

In quantum mechanics, the uncertainty principle does not allow us to give meaning to the ‘trajectory’ of a single particle so we are left with the question: “How does a particle get from *A* to *B*?”. Rather than taking two points, consider two small volumes, $\Delta V^{\prime }\left(x^{\prime }\right)$ surrounding the point $A=x^{\prime }$ and ΔV(x) surrounding B=x. We assume these volumes are initially large enough to avoid problems with the uncertainty principle.

Now imagine a sequence of particles emanating from $\Delta V^{\prime }\left(x^{\prime }\right)$, each with a different momentum. Over time we will have a spray of possible momenta emerging from the volume $\Delta V^{\prime }\left(x^{\prime }\right)$, the nature of this spray depending on the size of $\Delta V^{\prime }\left(x^{\prime }\right)$. Similarly there will be a spray of momenta over time arriving at the small volume ΔV(x) surrounding the point *x*.

**Figure 1:**
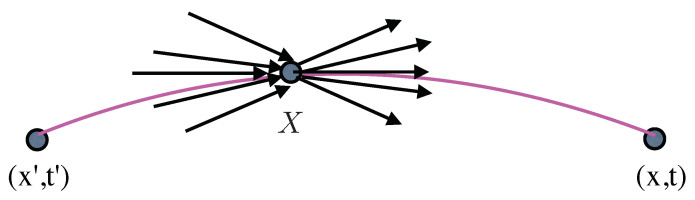
Behaviour of the momenta sprays at the midpoint of ${\langle x,t|x^{\prime },t^{\prime }\rangle }_{\epsilon }$.

Better still let us consider a small volume surrounding the midpoint *X*. At this point there is a spray arriving and a spray leaving a volume ΔV(X) as shown in Figure 1. To see how the local momenta behave at the midpoint *X*, we will use the real part of $S_{\epsilon }^{\prime }\left(x,x^{\prime }\right)$ defined by

(14) $$\begin{array}{c} S_{\epsilon }\left(x,x^{\prime }\right)=\frac{m}{2}\frac{{\left(x-x^{\prime }\right)}^{2}}{\epsilon }. \end{array}$$

Let us define a quantity

(15) $$\begin{array}{c} P_{X}\left(x,x^{\prime }\right)=\frac{\partial S_{\epsilon }\left(x,x^{\prime }\right)}{\partial X}=\frac{\partial S_{\epsilon }\left(X,x^{\prime }\right)}{\partial X}+\frac{\partial S_{\epsilon }\left(x,X\right)}{\partial X}, \end{array}$$

then using the action (Equation (14)), we find

(16) $$P_{X}\left(x,x^{\prime }\right)=m\left[\frac{\left(X-x^{\prime }\right)}{\epsilon }-\frac{\left(x-X\right)}{\epsilon }\right]=p_{X}^{\prime }\left(x,x^{\prime }\right)+p_{X}\left(x,x^{\prime }\right).$$

Not surprisingly, this is exactly what Feynman [9] is averaging over at the point *X*, agreeing with the term between the brace […].

What is more important is the relation of Equation (16) to Equation (10) which is the real part of the weak value of the momentum operator. Thus, the mean momentum of a set of Feynman paths at *X* is clearly the real part of this weak value. However, this weak value is just the Bohm momentum. Thus the Bohm ‘trajectories’ are simply an ensemble of the average of the ensemble of individual Feynman paths.

To see how this unexpected result also emerges from a different perspective, let us consider the process in Figure 1 which we regard as an image of an ensemble of actual individual quantum processes. We are interested in finding the average behaviour of the momentum, $P_{X}$, at the point *X*. However, we have two contributions to consider, one coming from the point $x^{\prime }$ and one leaving for the point *x*. We must determine the distribution of momenta in each spray to produce a result that is consistent with the wave function ψ(X) at *X*. Feynman suggests [9] that we can think of ψ(X) as ‘information coming from the past’ and $\psi^{*}\left(X\right)$ as ‘potential information appearing in the future’. This suggests that we can write

$$\begin{array}{c} \lim_{x^{\prime }\to X}\psi \left(x^{\prime }\right)=\int \phi \left(p^{\prime }\right)e^{ip^{\prime }X}dp^{\prime }\;\mathrm{and}\;\lim_{X\to x}\psi^{*}\left(x\right)=\int \phi^{*}\left(p\right)e^{-ipX}dp. \end{array}$$

The $\phi (p^{\prime })$ contains information regarding the probability distribution of the incoming momentum spray, while $\phi^{*}\left(p\right)$ contains information about the probability distribution in the outgoing momentum spray. These wave functions must be such that in the limit ϵ→0 they are consistent with the wave function ψ(X).

Thus, we can define the mean momentum, $\bar{\bar{P}}\left(X\right)$, at the point *X* as

(17) $$\begin{array}{c} \rho \left(X\right)\bar{\bar{P}}\left(X\right)=\int \int P\phi^{*}\left(p\right)e^{-ipX}\phi \left(p^{\prime }\right)e^{ip^{\prime }X}\delta \left(P-\left(p^{\prime }+p\right)/2\right)dPdpdp^{\prime } \end{array}$$

where ρ(X) is the probability density at *X*. We have added the restriction $\delta (P-\left(p^{\prime }+p\right)/2)$ since momentum is conserved at *X*. We can rewrite Equation (17) and form

$$\begin{array}{c} \rho \left(X\right)\bar{\bar{P}}\left(X\right)=\frac{1}{2\pi }\int \int P\phi^{*}\left(p+\theta /2\right)e^{-iX\theta }\phi \left(p-\theta /2\right)d\theta dP \end{array}$$

or equivalently taking Fourier transforms

$$\begin{array}{c} \rho \left(X\right)\bar{\bar{P}}\left(X\right)=\frac{1}{2\pi }\int \int P\psi^{*}\left(X-\sigma /2\right)e^{-iP\sigma }\psi \left(X+\sigma /2\right)d\sigma dP \end{array}$$

which means that $\bar{\bar{P}}\left(X\right)$ is the conditional expectation value of the momentum weighted by the Wigner function. Equation (17) can be put in the form

(18) $$\begin{array}{c} \rho \left(X\right)\bar{\bar{P}}\left(X\right)=\left(\frac{1}{2i}\right){\left[\left(\partial_{x_{1}}-\partial_{x_{2}}\right)\psi \left(x_{1}\right)\psi \left(x_{2}\right)\right]}_{x_{1}=x_{2}=X} \end{array}$$

an equation that appears in the Moyal approach [30], which is based on a different non-commutative algebra. If we evaluate this expression for the wave function written in polar form ψ(x)=R(x)exp[iS(x)], we find $\bar{\bar{P}}\left(X\right)=\nabla S\left(X\right)$ which is identical to the expression for the local (Bohm) momentum used in the Bohm interpretation.

This then confirms the conclusion we reached above, namely, that the set of Bohm ‘trajectories’ is an ensemble of the average ensemble of individual paths. Notice, once again, that this gives a very different picture of the Bohm momentum from the usual one used in Bohmian mechanics [31]. It is not the momentum of a single ‘particle’ passing the point *X*, but the mean *momentum flow* at the point in question.

This conclusion is supported by the experiments of Kocsis et al. [5]. They construct the flow lines from an average made over many individual input photons. Thus, the so-called ‘photon’ flow-lines are constructed *statistically* from an ensemble of individual events. As was shown in Flack and Hiley [26], these flow lines are an average of the momentum flow as described by the *weak* value of the Poynting vector. This agrees with what one would expect from standard quantum electrodynamics, where the notion of a ‘photon trajectory’ has no meaning, but the notion of a ‘momentum flow’ does have meaning.

**Figure 2:**
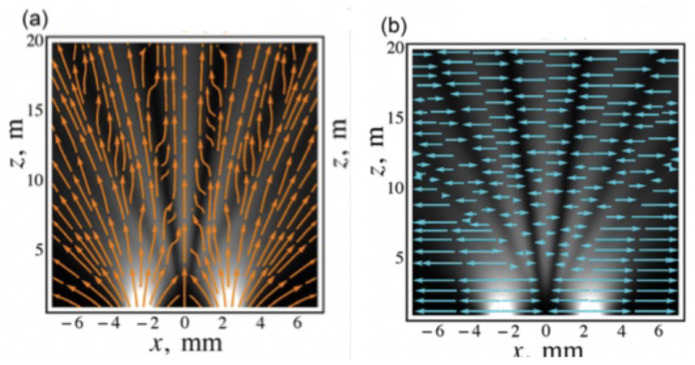
(**a**) Local field momentum; (**b**) Localising field momentum.

Bliokh et al. [32] have presented a beautiful illustration showing the results of a two-slit interference experiment. Figure 2a shows the real part of the momentum flow lines in the electromagnetic field, while the imaginary component (osmotic) momentum flow lines are shown in Figure 2b. It is then clear that we can regard $v_{B}\left(x,t\right)=p_{B}\left(x,t\right)/m$ as a *local* velocity, while the osmotic velocity $v_{O}\left(x,t\right)=p_{O}\left(x,t\right)/m$ can be regarded as a *localising* velocity as discussed in Bohm and Hiley [33]. The osmotic velocity behaves in such a way as to maintain the form of the probability distribution.

### 3.2. Where Is the Quantum Potential?

One of the features that many find ‘mysterious’ [34] is the appearance of the ‘quantum potential’ in the Bohm approach. Is there any trace of it in the Feynman paper [9]? To answer this question, we must first refer to de Gosson and Hiley [18] where it is shown that this energy term is absent in quantum processes when taken only to O(Δt=ϵ) so we must consider terms to $O(\Delta t=\epsilon^{2})$.

Feynman shows that the kinetic energy is of $O(\epsilon^{2})$ when written in the form $K.E.={\left[\left(x_{k+1}-x_{k}\right)/\epsilon \right]}^{2}$, and diverges as ϵ→0. Feynman points out that this quantity is not an observable functional. However, let us now define the kinetic energy to be

$$\begin{array}{c} {K.E.}^{\prime }=\frac{m}{2}\left(\frac{x_{k+1}-x_{k}}{\epsilon }\right)\left(\frac{x_{k}-x_{k-1}}{\epsilon }\right). \end{array}$$

This function is finite to O(ϵ) and therefore is an observable functional. Feynman then shows that if we allow “the mass to change by a small amount to m(1+δ) for a short time, say ϵ around $t_{k}$” we can obtain the relation

(19) $$\begin{array}{c} \frac{m}{2}\left(\frac{x_{k+1}-x_{k}}{\epsilon }\right)\left(\frac{x_{k}-x_{k-1}}{\epsilon }\right)=\frac{m}{2}{\left(\frac{x_{k+1}-x_{k}}{\epsilon }\right)}^{2}+\frac{\hbar }{2i\epsilon }, \end{array}$$

the extra term arising from the normalising function *A*. Thus, we must add a ‘correction’ term to the K.E. in order for the total energy to be finite to $O(\epsilon^{2})$.

This is the forerunner of mass renormalisation used in quantum electrodynamics. In that case the charged particle is subjected to electromagnetic vacuum fluctuations. The particle we are considering here is not charged and so the fluctuation must arise from a different source, but however it arises, it changes the TPA by δ.

Later in the same paper, Feynman shows that any random fluctuation in the phase function will produce the same effect. A random fluctuation at the point $x_{k}$ implies we must replace $S(x_{k+1},t_{k+1};x_{k},t_{k})$ by $S_{\delta }\left(x_{k+1},t_{k+1};x_{k},t_{k}-\delta \right)$. Thus, to the first order in δ we have

$$\begin{array}{c} {\langle \xi |1|\psi \rangle }_{S}-{\langle \xi |1|\psi \rangle }_{S_{\delta }}=\frac{i\delta }{\hbar }\langle \xi |H_{k}{|\psi \rangle }_{S} \end{array}$$

where $H_{k}$ is the Hamiltonian functional

(20) $$\begin{array}{c} H_{k}=-\frac{\partial S(x_{k+1},t_{k+1};x_{k},t_{k})}{\partial t_{k+1}}+\frac{\hbar }{2i(t_{k+1}-t_{k})}. \end{array}$$

Apart from the minus sign, the last term is identical to the last term in Equation (19). Thus Feynman required extra energy to appear from somewhere. A more detailed discussion of this feature appears in Feynman and Hibbs [35]. The Bohm approach indicates that some ‘extra’ energy appears in the form of the quantum potential energy at the expense of the kinetic energy. Could it be that the source of the energy is the same?

To explore this possibility, let us use the method explained in Section 2.3 to obtain a more general result for the K.E. The real part of the weak value of the momentum operator squared is obtained from $\left(\langle \psi \left(t\right)|{\hat{p}}^{2}|x\rangle +\langle x|{\hat{p}}^{2}|\psi \left(t\right)\rangle \right)/2$. Under polar decomposition of the wave function, we find the real part of the weak value of the kinetic energy is

(21) $$\begin{array}{c} \frac{1}{2m}{\langle {\hat{p}}^{2}\rangle }_{w}=\frac{1}{2m}\left[{\left(\nabla S\right)}^{2}-\frac{\nabla^{2}R}{R}\right]. \end{array}$$

With the identification $\nabla S↔m(x_{k+1}-x_{k})/\epsilon$, we see that the quantum potential is playing a similar role as the mass/energy fluctuation in Feynman’s approach. In fact, de Broglie’s original suggestion was that the quantum potential could be associated with a change of the rest mass [36].

Notice that the quantum potential appears essentially as a derivative of the osmotic velocity, which in turn is obtained from the imaginary part of $S^{\prime }\left(x,x^{\prime }\right)$. Any fluctuating term added to the real part of $S_{\epsilon }\left(x,x^{\prime }\right)$ should also be added to the imaginary part. This would also introduce some change in the energy relation shown in Equation (20). This interplay between the real components of the complex $S_{\epsilon }\left(x,x^{\prime }\right)$ is clearly presented as an average over fluctuations arising from some background. Here we can recall Bohr insisting that quantum phenomena must include a description of the whole experimental arrangement. More details will be found in Smolin [37] and in Hiley [38].

## 4. Conclusions

Our explorations of the weak values of the momentum operator [22] have led us to reconsider the basis on which Feynman [9] developed his path integral approach. We have shown that there is an unexpected close connection between the Feynman propagator, the weak values of the momentum and the original Bohm approach [13].

Feynman had already noticed that to prevent the kinetic energy tending to infinity as the time interval between steps tends to zero, it was necessary to introduce a ‘fluctuation’ in the mass/energy of the particle. This extra energy can be thought of as arising in a way similar to the way the quantum potential energy appears as an effect of some background field. Indeed, as we have remarked above, de Broglie [36] had already proposed that the quantum potential could be included in the mass term $M=\surd [m^{2}+\left(\hbar^{2}/c^{2}\right)□R/R]$, *R* being the amplitude of the wave function. Hiley [38] has shown a similar conclusion arises for the Dirac equation.

The approach outlined in this paper shows that the basic assumption made in Bohmian mechanics, namely, that each particle follows one of the ensemble of ‘trajectories’ calculated by Philippidis et al. [6] from $P_{B}\left(x,t\right)$ cannot be maintained. Rather the trajectories should be interpreted as a statistical average of the momentum flow of a basic underlying stochastic process.

It is now possible to experimentally explore weak values, perhaps clarifying the nature of this stochastic process. In the case of the electromagnetic field this has already been done by Kocsis et al. [5], but as we have seen the notion of a ‘photon trajectory’ has no meaning. However, the average momentum flow does have meaning [26]. As mentioned above, new experiments using argon and helium atoms are now being carried out at UCL by Morley et al. [29] and by Monachello, Flack, and Hiley [28]. It is hoped that these future experiments will throw more light on the nature of individual quantum processes.
